# Assessment of genetic diversity and phylogenetic relationship of Limousin herds in Hungary using microsatellite markers

**DOI:** 10.5713/ajas.18.0164

**Published:** 2018-07-26

**Authors:** Márton Szűcs, Ferenc Szabó, Beáta Bán, Csilla Józsa, László Rózsa, Attila Zsolnai, István Anton

**Affiliations:** 1 Association of Hungarian Limousin and Blonde d’Aquitaine Breeders, Lőportár u. 16., Budapest, 1134, Hungary; 2 Department of Animal Sciences, Széchenyi István University, Vár tér 2., Mosonmagyaróvár, 9200, Hungary; 3 National Foodchain Safety Office, Tábornok u. 2., Budapest, 1143, Hungary; 4 NARIC-Research Institute for Animal Breeding Nutrition and Meat Science, Gesztenyés u. 1., Herceghalom, 2053, Hungary

**Keywords:** Limousin Cattle, Microsatellite, Genetic Diversity, Genetic Information

## Abstract

**Objective:**

This study was conducted to investigate basic information on genetic structure and characteristics of Limousin population in Hungary. Obtained results will be taken into consideration when adopting the new breeding strategy by the Association of Hungarian Limousin and Blonde d’Aquitaine Breeders (AHLBB).

**Methods:**

Genetic diversity and phylogenetic relationship of 3,443 Limousin cattle from 16 different herds were investigated by performing genotyping using 18 microsatellite markers. Amplified DNA was genotyped using an automated genetic analyzer.

**Results:**

Mean of effective alleles (n_e_) of the populations was 3.77. Population C had the lowest number of effective alleles (3.01) and the lowest inbreeding coefficient (F_IS_) value (−0.15). Principal component analysis of estimated genetic distance (F_ST_) values (p<0.000) revealed two herds (C and E) distinct from the majority of other Limousin herds. The pairwise F_ST_ values of population C compared to the others (0.066 to 0.120) fell into the range of moderate genetic distance: 0.050 to 0.150, while population E displayed also moderate genetic distance (F_ST_ values in range 0.052 to 0.064) but only to six populations (G, H, J, L, N, and P). F_ST(C-E)_ was 0.148, all other pairs -excluding C and E herds- displayed low genetic distance (F_ST_<0.049). Population D, F, I, J, K, L, N, O, and P carried private alleles, which alleles belonged to 1.1% of the individuals. Most probable number of clusters (K) were 2 and 7 determined by Structure and BAPS software.

**Conclusion:**

This study showed useful genetic diversity and phylogenetic relationship data that can be utilized for the development of a new breeding strategy by AHLBB. The results presented could also contribute to the proper selection of animals for further whole genome scan studies of Limousins.

## INTRODUCTION

Beef consumption is related to living standards, diet, livestock production, consumer prices and is dependent on either cultures or religions. In South America beef is the favorite type of meat. Uruguay consumed the most beef per capita in the world in 2017, followed by Argentina. Both countries consumed more than 40 kg of beef per capita [1]. Meat consuming habits of Hungarian consumers greatly differ from those of other EU citizens and South American customers. Most popular types of meat in Hungary are poultry and pork meat, whereas beef consumption has fallen to 2.5 kg per capita [2].

This is why primary goal of Hungarian cattle breeders is to produce a top-quality beef that consumers are willing to purchase, a tender, juicy and flavourful product of good value. Since its foundation in 1989, the Association of Hungarian Limousin and Blonde d’Aquitaine Breeders (AHLBB) has been taking measures to improve quality of beef and to meet demand of consumers with high quality products, by introducing a strict performance testing, qualification and selection program.

Since the establishment of Limousine breed (1886), about 70 countries imported significant number of Limousine cattle for breeding [3] because the body composition and saleable meat yield (73.3%) are favourable and meets the demands of the market [4].

The highly polymorphic microsatellite markers are widely used as genetic markers for purposes that include population genetics, parentage identification, fingerprinting, genetic mapping and conservation [5,6]. Microsatellite population studies are more frequently applied on local breeds e.g. in China [7], Oman [8], Korea [9] to clarify origin or position of these breeds relative to other ones. Population study can also be performed within a breed [10] to clarify herd position and highlight those populations which require attention by the management. Since studies concerning the genetic diversity and phylogenetic relationship of Limousin cattle on a global scale are few in number [11,12] and are completely lacking in Hungary, it has been decided to carry out studies within the existing Limousin population with the aim to provide additional data to this particular subject.

## MATERIALS AND METHODS

**Figure 1:**
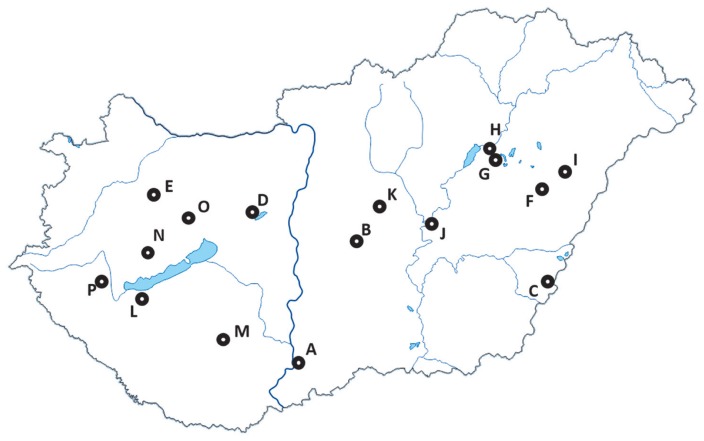
Sampling locations of the 16 Limousin herds.

Sixteen different Limousin cattle herds, maintained for commercial use, were included in this study. Blood samples were collected for routine parentage testing, by breeders during their established breeding program, from jugular veins from 3,443 individuals (1,520 bulls and 1,924 cows) in tubes containing ethylenediaminetetraacetic acid. Samples were stored at −20°C until genomic DNA extraction, which was performed using the QIAamp DNA Mini Kit (QIAGEN, Hilden, Germany). All data concerning registration of herds and codes used in this study were provided by AHLBB. Sampling locations are presented on Figure 1.

All 18 microsatellite markers used herein (BM1818, BM 1824, BM2113, CSRM60, CSSM66, ETH10, ETH225, ETH3, ILSTS006, INRA23, MGTG4B, RM067, SPS113, SPS115, TGLA 122, TGLA126, TGLA227, TGLA53) are recommended by International Society of Animal Genetics (ISAG) for routine parentage control and record exchange between laboratories [13]. Polymerase chain reaction (PCR) conditions were applied according to instruction manual of Bovine Genotypes Panel 1.1 (Finnzyme Diagnostics, Keilaranta, Finland), whereas the PCRs were performed on an ABI 9700 PCR system (Applied Biosystems, Foster City, CA, USA). Fragment length determination was accomplished on an automated ABI 3100 Genetic Analyzer (Applied Biosystems, USA) according to the manufacturer’s instructions.

Statistical analysis: Exact test of Hardy-Weinberg equilibrium (HWE) and exact test of population differentiation were calculated by Genepop 4.2.1 [14], FSTAT 2.9.3.2 [15], and Arlequine [16] programmes. Estimation of exact P value of Hardy-Weinberg test was performed on each locus and each population. Evidence for the presence of null alleles at each locus was evaluated using Genepop and Micro-Checker version 2.2.3 (Monte Carlo simulation; bootstrap method) [17]. Observed heterozygosity (Ho), expected heterozygosity (He), inbreeding coefficient (F_IS_), genetic distance (F_ST_) indices were calculated by Genalex 6.5 [18]. Data were crosschecked by FSTAT and Genepop.

Bayesian algorithm implemented in Structure was used for inferring the most probable number of clusters (K) (burn-in: 10^5^, MCM steps: 5×10^5^, repetition: 5, model: admixture, allele frequencies correlated) and for calculation of membership probability of individuals. For estimation of K, Evanno’s method [19] was applied on Structure output. Bayesian stochastic partition-based approach implemented in BAPS 6.0 [20] was also applied to estimate K.

A weighted principal component analysis (PCA) was performed using the allele frequency data of Limousin individuals and the 18 microsatellite markers using Genalex.

Nei’s genetic distance was calculated and viewed by Poptree software [21]. Bootstrap values were based on 1,000 permutations.

Assignment tests of individuals were performed by Genalex and Geneclass 2.0 softwares [22] using a Bayesian method [23, 24] and a simulation algorithm [19] with 10,000 simulated individuals.

## RESULTS AND DISCUSSION

Thirty eight of the 288 chi-square tests showed significant deviations from HWE at the 95% confidence interval. Heterozygote excess was calculated in each herd, the highest excess was detected in herd C. Allelic richness ranged between 4.31 and 5.21 (population C and O, respectively). Private alleles were detected in nine herds. Altogether 38 animals (1.1% of the analysed individuals) carried one or more private alleles (Table 1). Herd characteristics (Table 1) and diversity information of the microsatellite loci (Table 2) are similar to the values reported by Amigues et al [11] and Radko et al [12] on Limousin cattle of France and Poland. In our study the number of private alleles (PA = 20) was higher whereas the number of effective alleles was similar (3.8 vs 4) to those obtained by Amigues et al [11] (PA = 6). The difference in PA might account for the higher number of investigated animals herein.

In genetic assignment test (data not shown) 48% of the animals have been allocated correctly to their original groups. In more detail the corresponding values were 90% and 100% in the herds E and C, supporting that these herds are more different from the others. Cumulative exclusion probability value was higher than 0.999 in accordance to the results obtained by Radko et al [12].

Pairwise exact genotypic differentiation tests performed by FSTAT and Genepop showed that—except the pair B, K—all the herds can be treated as separate units, distinct from each other (p<0.05).

**Figure 2:**
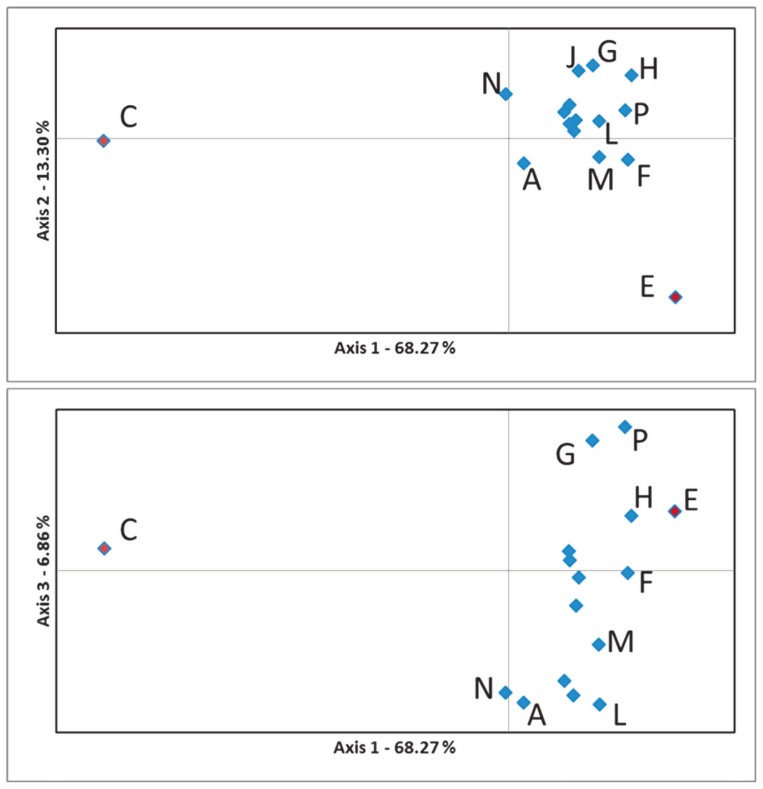
Representation of principal component analysis (PCA) of estimated pairwise FST values obtained by Genalex software. Blue labelled herds are in the range of low genetic differentiation. Red labelled herd C have moderate genetic distance from the other populations, while herd E moderately differentiated from G, H, J, L, N, and P populations. Percentage values represent variation justified by each axis.

Consecutive PCA analysis of estimated F_ST_ values (p<0.000, Table 3.) revealed two herds (C and E) distinct from the majority of other Limousin herds (Figure 2). The pairwise F_ST_ values of population C compared to the others (0.066 to 0.120) fell into the range of moderate genetic distance: 0.050 to 0.150 [25], while population E displayed also moderate genetic distance (F_ST_ values in range 0.052 to 0.064) but only to six populations (G, H, J, L, N, and P). F_ST(C-E)_ was 0.148, all other pairs—excluding C and E herds—displayed low genetic distance (F_ST_<0.049).

**Figure 3:**
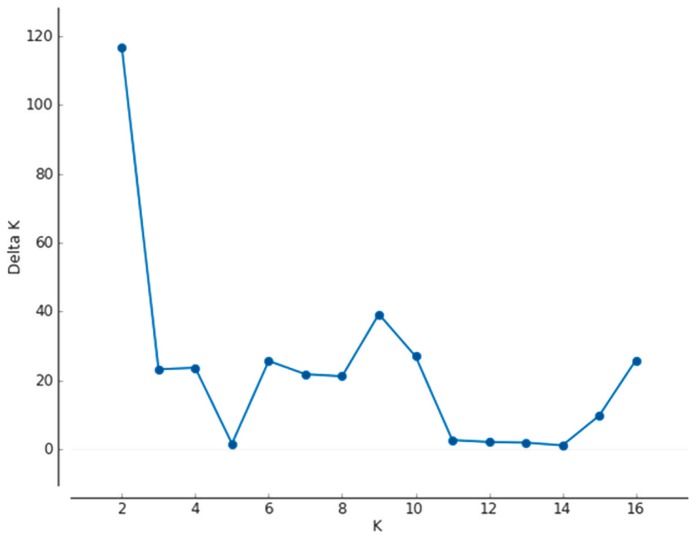
Determination of the most probable cluster number of 16 Limousin herds using ΔK approach on Structure lnP(D) values. ΔK values (five independent runs) for each assessed K value on 16 Limousin populations. The most probable number of clusters was two.

Structure programme revealed that the most probable number of clusters among 16 Limousine herds was two (K_Evanno_ = 2, Figure 3). Structure indicated only two major groups, where population A, C, N were separated from the remaining 13 populations.

**Figure 4:**
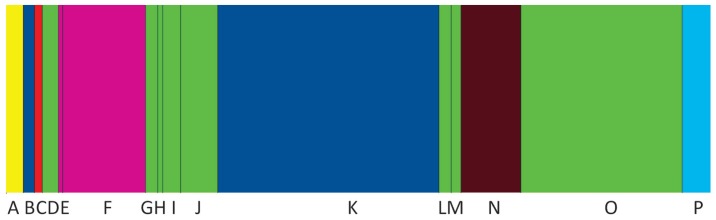
Clustering of Limousin herds by BAPS software. Clusters consisting more than one groups are: B, K (blue), D, G, H, I, J, L, M, O (green), E, F (cyclamen). Horizontal widths of the rectangles are proportional to the number of individuals genotyped within a herd. Group boundaries are vertical black lines.

Clustering of populations showed K = 7 calculated by BAPS software, where populations A, C, N, and P belonged to distinguished clusters, while the remaining three groups were formed by herds B-K, E-F, and D-G-H-I-J-L-M-O, respectively (Figure 4).

**Figure 5:**
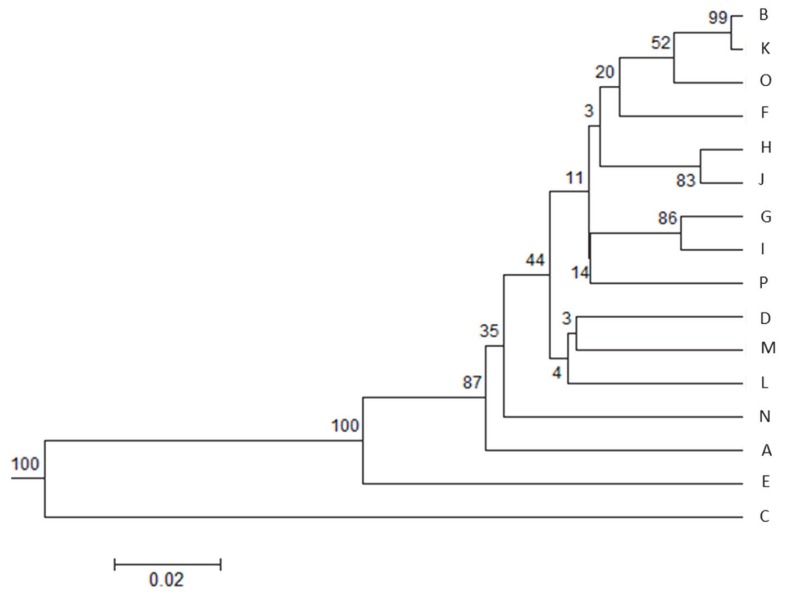
UPGMA tree of Nei’s genetic distance of Limousin cattle from 16 farms. Bootstrap values are indicated on the nodes.

UPGMA tree of Nei’s genetic distance (Figure 5) of populations showed the above mentioned same three (A, C, N) herds as distinct groups, but instead of herd P (identified by BAPS) the population E was placed on a distinct branch. This latter observation agrees with the PCA analysis of F_ST_ values. Population B and K remained together on the dendrogram. The remaining groups are the least divergent from each other. Plotting neighbour joining tree of genetic distance (data not shown) the A, C, N populations remained separated. E herd was more similar to the remaining herds and it shared a node with population F -as we see on the BAPS generated result (Figure 4) but was placed on the longest branch among populations (excluding A, C, N) in accordance with the PCA analysis.

In case of herd A, C, N, and E it is known from the herd books, that cows, semen or embryos have been imported from different regions of France which explain the differences visible mostly on Figure 2, 5. In case of populations C and E the extent of imported individuals were higher than that of the population of A and N.

## CONCLUSION

Based on the outcome of this study, we recommend the cautious use of individuals of population C and E in the new breeding strategy since their FST distance to the other herds are already in a moderate range. Private alleles, which are recommended to be preserved in populations are found in 9 farms (D, F, I, J, K, L, N, O, and P) which should also be taken into consideration in the breeding plans of the AHLBB.

## Figures and Tables

**Table 1:** Statistical analysis of 16 Limousin cattle herds

Herd code	n	AR	n_e_	PA	n_PA_	Ho	He	F_IS_
A	85	4.80	3.73±0.19			0.77±0.02	0.72±0.01	−0.06±0.02*
B	54	5.11	3.90±0.23			0.75±0.02	0.73±0.01	−0.03±0.01*
C	37	4.31	3.01±0.25			0.71±0.04	0.62±0.03	−0.15±0.02
D	79	4.84	3.87±0.32	1	7	0.75±0.02	0.71±0.02	−0.05±0.02*
E	21	4.69	3.55±0.20			0.74±0.02	0.70±0.02	−0.05±0.02
F	404	4.80	3.65±0.23	4	11	0.73±0.02	0.71±0.02	−0.03±0.01
G	58	4.85	3.59±0.23			0.72±0.03	0.70±0.02	−0.02±0.03
H	25	5.04	3.84±0.23			0.76±0.03	0.72±0.02	−0.05±0.03*
I	87	5.13	3.94±0.24	2	3	0.75±0.02	0.73±0.02	−0.02±0.01
J	180	5.28	4.14±0.32	1	2	0.74±0.02	0.73±0.02	0.00±0.01*
K	1076	5.18	4.01±0.22	6	5	0.73±0.01	0.74±0.01	0.01±0.01
L	60	5.11	3.77±0.34	2	3	0.71±0.03	0.70±0.02	−0.01±0.02
M	48	4.86	3.83±0.29			0.74±0.02	0.72±0.02	−0.03±0.02
N	291	5.04	3.94±0.26	1	3	0.74±0.02	0.73±0.02	−0.02±0.01
O	786	5.21	4.08±0.28	2	3	0.74±0.02	0.74±0.02	0.00±0.00*
P	152	4.67	3.47±0.25	1	1	0.70±0.03	0.68±0.02	−0.02±0.01

n, number of individuals; AR, allelic richness; n_e_, number of effective alleles; PA, number of private alleles; n_PA_, number of animals with one or more private alleles; Ho, average observed heterozygosity (mean±standard error); He, average expected heterozygosity (mean±standard error);

* F_IS_, inbreeding coefficient values did not differ significantly from zero after bootstrapping (confidence interval = 0.95).

**Table 2:** Number of alleles (N), observed and expected heterozygosity (Ho and He), polymorphism information content (PIC), and F-statistics values of 18 microsatellite markers among 16 Limousin cattle breeds

Locus	N	Ho	He	PIC	F_ST_	F_IT_	F_IS_
BM1818	7	0.672	0.671	0.627	0.034	0.019	−0.016
BM1824	4	0.686	0.672	0.609	0.034	−0.028	−0.065
BM2113	10	0.806	0.803	0.776	0.044	0.007	−0.039
ETH3	9	0.745	0.717	0.675	0.035	−0.040	−0.077
ETH10	7	0.714	0.740	0.700	0.073	0.044	−0.031
ETH225	7	0.714	0.704	0.648	0.049	−0.021	−0.074
INRA23	10	0.774	0.783	0.750	0.039	0.003	−0.037
SPS115	8	0.718	0.722	0.679	0.042	−0.005	−0.049
TGLA53	18	0.814	0.829	0.811	0.057	0.038	−0.020
TGLA122	15	0.794	0.799	0.774	0.052	0.010	−0.044
TGLA126	7	0.628	0.635	0.585	0.054	0.004	−0.052
TGLA227	15	0.842	0.831	0.808	0.043	−0.018	−0.064
CSRM60	9	0.693	0.708	0.671	0.045	0.044	−0.001
CSSM66	12	0.820	0.826	0.805	0.038	0.008	−0.032
ILSTS006	10	0.669	0.694	0.655	0.043	0.032	−0.011
MGTG4B	12	0.674	0.686	0.660	0.040	0.039	−0.001
RM067	9	0.678	0.681	0.638	0.060	0.024	−0.038
SPS113	11	0.839	0.831	0.809	0.034	−0.008	−0.043
Mean	10	0.7378	0.7407	0.7044	0.0453	0.0084	−0.039

**Table 3:** Pairwise F_ST_ values of the studied populations marked by letters

Items	A	B	C	D	E	F	G	H	I	J	K	L	M	N	O
B	0.026														
C	0.078	0.091													
D	0.034	0.024	0.097												
E	0.049	0.044	0.148	0.051											
F	0.041	0.025	0.117	0.030	0.038										
G	0.048	0.028	0.110	0.041	0.064	0.044									
H	0.049	0.026	0.120	0.032	0.059	0.031	0.025								
I	0.023	0.012	0.087	0.032	0.044	0.026	0.009	0.021							
J	0.038	0.025	0.098	0.028	0.060	0.032	0.021	0.007	0.016						
K	0.032	0.010	0.090	0.029	0.045	0.024	0.028	0.027	0.017	0.027					
L	0.036	0.026	0.108	0.023	0.052	0.030	0.042	0.032	0.028	0.028	0.032				
M	0.028	0.021	0.104	0.024	0.035	0.027	0.036	0.033	0.022	0.033	0.027	0.026			
N	0.029	0.024	0.066	0.034	0.063	0.044	0.038	0.039	0.018	0.030	0.031	0.026	0.030		
O	0.024	0.019	0.087	0.018	0.049	0.019	0.033	0.024	0.019	0.022	0.025	0.016	0.023	0.019	
P	0.048	0.025	0.119	0.044	0.055	0.027	0.030	0.027	0.021	0.035	0.028	0.036	0.034	0.046	0.033
